# Oral Microbiota: Discovering and Facing the New Associations with Systemic Diseases

**DOI:** 10.3390/pathogens9040313

**Published:** 2020-04-24

**Authors:** Romeo Patini

**Affiliations:** Department of Head, Neck and Sense Organs, School of Dentistry, A. Gemelli Teaching Hospital Foundation—IRCCS, Catholic University of Sacrted Heart, 00168 Rome, Italy; romeo.patini@unicatt.it

**Keywords:** oral microbiota, systemic diseases, microbiology, clinical trial, systematic review

## Abstract

The economic crisis of the first decades of the 2000s had serious repercussions on the economy of individual countries, producing a gradual impoverishment of populations. The reduction in financial resources has significantly reduced citizens’ access to care, forcing them to abandon preventive medicine treatments and check-ups. The health of the oral cavity, which had long been considered of secondary importance when compared with systemic pathologies whose course can be potentially fatal for the patient, has therefore been strongly neglected. In recent years, however, new mechanisms of etiology of systemic diseases have been studied with the aim of evaluating some aspects still unknown. The microbiota, whose interest has grown considerably in the national scientific community, was immediately considered as a key factor in the pathogenesis of some disorders. These analyses have also benefited from numerous advances in the field of crop and molecular diagnostics in the microbiological field. Although pioneering studies have focused on the microbiota of the gastro-intestinal system, subsequent evidence has also been drawn from various studies conducted on the oral microbiota. What emerged is that oral microbiota dysbiosis has been associated with numerous systemic diseases. Therefore, the purpose of this Special Issue is to encourage scientific research on the topic of the relationship between the oral microbiota and systemic diseases, also inviting the use of new techniques for culture and molecular diagnosis. Particular attention will be given to original works in vivo and to literature reviews provided they are carried out with a systematic approach and, if possible, supported by additional quantitative analyses.

The term “microbiota” expresses the concept of all bacterial species, which form the bacterial community within a particular biological fluid, organ or system [1]. Even if the bacterial kingdom is the most investigated in literature, the microbiota also includes archaea, fungi, protists and viruses. Such a term should not be confused with the term “microbiome” that represents the set of genomes of microorganisms resident in humans [2].

The first research on the microbiota has focused mainly on the gastro-intestinal tract, revealing unpredictable connections with other organs and systems, even so far as the central nervous system [3,4,5,6].

In recent years, however, research has shifted its interest also to the microbiota of other biological fluids and organs, like the oral cavity and saliva or crevicular fluid, attempting to discover new associations with some systemic diseases with the aim of developing new treatment strategies.

As is well known, the oral cavity is a potential biological niche for bacterial colonization. In 2016, in fact, it was discovered that over 700 species could inhabit the human mouth and of these over 30% were unknown. Other authors have also reported that over 300 different bacterial species can colonize the oral cavity of a healthy individual [7]. 

After the discovery of the great variety that characterizes the oral microbiota (OM), a high number of studies have focused on the relationships between OM and systemic diseases [8,9,10,11,12,13,14,15,16,17].

The main areas of interests have been: central nervous system (CNS) diseases, gastro-intestinal diseases, autoimmune diseases and oncological diseases.

Almost all of the published articles are cross-sectional studies in which it was desired to investigate the composition of the oral microbiota in order to identify one or more microorganisms to be correlated to a specific pathology. Most reports analyze the microbiota of saliva or bacterial plaque [18,19,20], but some publications, especially in the field of oncology, also evaluate the microbiota on biopsies tissues [21].

Once the presence of specific pathogens had been ascertained in patients with certain diseases, the authors attempted to define the pathogenesis mechanisms by which the microorganisms that make up OM would support these pathologies. Therefore, CNS diseases seem to be sustained by the pathogen action of bacteria belonging to the genus *Actinomyces* that are able to alter synapse function and microglial activity, and belonging to the genus *Clostridium* and *Spirochaetes* that can alter the CNS metabolism through the degradation or production of some amino acids [22]. On the other hand, gastro-intestinal disorders seem to be mediated by the interference with signaling pathways and by the inhibition of the immune response realized by bacteria; moreover, oncological diseases (among which gastro-intestinal ones are also included) are sustained by the chronic inflammation response that can promote the progression of the cancer [23]. Besides this, other reports revealed that OM dysbiosis could also induce the increase of onco-epigenetic alterations or promote the ulceration of the epithelial barrier up to the onset of precancerous lesions, even in very distant organs [24].

Some new approaches, which involve the use of microorganisms belonging to the commensal bacterial flora, have been presented for the treatment of infections resistant to some antibiotics. Among them, the fecal microbiota transplantation approach consists of the infusion of feces from a healthy donor to the gastrointestinal tract of a recipient patient to treat disease associated with alterations in gut microbiota, thus also reducing other systemic diseases associated with that dysbiosis [25].

In light of this, research in the dental field also needs further discoveries aimed at both a deeper knowledge of the OM and the treatment of its conditions of dysbiosis. The therapies of diseases related to dysbiosis, in fact, could go through the restoration of a symbiotic microbiota in the various organs, including the oral cavity. It has been well known for years that the stabilization and maintenance of oral microbiota is a key factor in oral health; now it is becoming equally known that OM is crucial also for general health, and researchers in dental fields are called to do their part.

For the abovementioned reasons, the final aim of this editorial and of the associated Special Issue is to spread the newest associations between OM and systemic diseases, along with new treatment strategies for the treatment of its dysbiosis.

The accepted topics include all branches of microbiology (from bacteriology to virology to mycology and parasitology); equally interesting are the manuscripts that present new OM collection and analysis techniques. Special interest will arouse research concerning new associations between OM and systemic diseases or wishing to reproduce designs already present in the literature in order to expand the existing sample. Multidisciplinary research is strongly encouraged, especially in the field of microbiology and infectious diseases.
